# Inversion and analysis of leaf area index (LAI) of urban park based on unmanned aerial vehicle (UAV) multispectral remote sensing and random forest (RF)

**DOI:** 10.1371/journal.pone.0320608

**Published:** 2025-03-24

**Authors:** Yan Li, Bocheng Wang, Xuefei Zhao, Yichuan Zhang, Lifang Qiao

**Affiliations:** 1 Department of Architecture, Henan Technical College of Construction, Zhengzhou, China; 2 School of Horticulture and Landscape Architecture, Henan Institute of Science and Technology, Xinxiang, China; Tennessee State University, UNITED STATES OF AMERICA

## Abstract

Leaf Area Index (LAI) is a critical indicator of vegetation growth and ecological function. Unlike the relatively uniform crop types and planting methods typically found in agricultural fields, parks typically feature a diverse range of plant species, varied configurations, and complex vertical structures, making LAI estimation more complex and challenging. To improve the accuracy of LAI estimation in urban parks, this study, by combining unmanned aerial vehicle (UAV) multispectral remote sensing technology with Random Forest (RF) to conduct the inversion and analysis of LAI in Xinxiang People’s Park. High-resolution images are obtained using multispectral sensors carried by a UAV, which are then used to calculate the Normalized Difference Vegetation Index (NDVI). Combined with ground-measured vegetation LAI data, this study applies RF to estimate the park LAI. The results indicate that the average LAI of Xinxiang People’s Park is 2.30 (for the entire park). excluding the hard surfaces (which account for 36.05%), the average LAI increases to 3.59, indicating good vegetation conditions. The LAI of the park and its distribution are influenced by factors such as plant species, configuration patterns, planting density, aesthetic design, and site function. Accurate LAI inversion is crucial for effective management and optimization of these green spaces. RF can effectively capture the complex nonlinear relationship between NDVI and LAI, with a coefficient of determination (R²) of 0.54 and a root mean square error (RMSE) of 0.91. Although the accuracy is still insufficient, RF’s ability to handle nonlinear relationships makes it an effective tool for LAI inversion in complex vegetation environments. LAI inversion of park vegetation based on UAV multispectral imagery can provide valuable insights for the management and optimization of park vegetation.

## Introduction

As a crucial green space in modern cities, parks not only provide a place for leisure and recreation for citizens, but also play a key role in regulating urban climate, improving air quality, and enhancing biodiversity, etc [1]. Normalized Difference Vegetation Index (NDVI) is a commonly used vegetation index for monitoring of vegetation health status, as it can intuitively reflect the status of vegetation growth [2]. Generally, a higher NDVI indicates greater vegetation coverage and better health, while a low NDVI may indicate low vegetation coverage or that the vegetation is affected by stresses such as drought, pollution, and others [3]. However, the two-dimensional information on vegetation coverage provided by NDVI cannot directly reflect three-dimensional structural characteristics, such as height, layers, and leaf density [4]. In contrast, Leaf Area Index (LAI) is a key parameter for measuring vegetation structure and ecological function, representing the total leaf area per unit ground area [5]. Unlike NDVI, LAI can reveal changes in vegetation height and density, which is of great significance for a deeper understanding of ecological processes in multi-layered vegetation systems, especially for evaluating complex ecosystems like the urban forest, etc [6].

As an important parameter for measuring vegetation canopy structure, LAI is closely related to the contribution of green spaces in providing ecosystem services. The ecological functions of vegetation can be comprehensively analyzed by studying the LAI of urban parks, providing an important basis for the management and optimization of green spaces. The cooling effect of green spaces is closely related to factors such as LAI value and canopy density, with areas having higher LAI values often exhibiting more significant cooling effects [7]. Therefore, the heat regulation function of green spaces can be significantly enhanced through rational planning and an increase in their LAI. Research has shown that when the LAI value of tree crown increases from 2 to 2.5, the impact of urban green spaces on reducing runoff is significantly enhanced [8]. In a study in Beijing, combining diameter at breast height, tree height, and LAI data, it was found that urban green spaces with higher LAI have a greater carbon storage capacity [9]. Additionally, the inversion of LAI from high-resolution satellite data can also be used for analyzing the dynamic changes in urban forests and provide a basis for formulating more targeted greening policies [10].

Extensive research has been conducted on using NDVI for LAI inversion, but most studies have focused on farmland [11], grasslands [12], or forests [13,14]. Compared to these ecosystems, park vegetation differs significantly (Table 1). Therefore, it is crucial to use appropriate high-resolution data and select a suitable model for LAI inversion in urban parks.

**Table 1:** Differences between park vegetation, farmland vegetation and forest vegetation.

Comparison dimension	Park vegetation	Farmland vegetation	Forest Vegetation
Vegetation structure & complexity	Predominantly artificial planting, diverse species (mix of trees, shrubs, and lawns), large canopy height variations, complex structure.	Primarily monoculture crops (e.g., wheat, corn), uniform canopy, simple structure, well-defined growth cycle.	Naturally diverse species, highly stratified with distinct canopy layers, structurally complex.
NDVI-LAI relationship	Mixed vegetation leads to NDVI being influenced by different species’ reflectance; machine learning models may be more effective.	Strong linear NDVI-LAI relationship for single crops; commonly uses empirical models (e.g., linear regression).	NDVI-LAI relationship varies due to dense canopy cover; saturation effect often occurs at high NDVI values.
Data acquisition method	Requires high spatial resolution data to distinguish fragmented and small-scale vegetation patches.	Medium-to-low resolution satellite data is sufficient for large-scale homogeneous farmland monitoring.	Requires high spectral and spatial resolution to capture vertical complexity and understory vegetation.
Interference factors	Human management, tourist activity, significant microclimate variations.	Farming cycles (sowing/harvesting), fertilizer/pesticide applications, periodic outbreaks of pests and diseases.	Natural disturbances (e.g., storms, wildfires, pests), ecological succession.

In recent years, the development of unmanned aerial vehicle (UAV) technology has provided strong technical support for LAI research in urban parks, demonstrating significant advantages. Traditional remote sensing technologies, especially low-resolution satellite images, often suffer from pixel-related issues, where a single pixel may contain multiple land types, leading to inaccurate LAI estimations. This problem is particularly evident in complex urban environments, where diverse ecosystems result in a significant decrease in estimation accuracy. In contrast, the flexibility and high-resolution data acquisition capabilities of UAV technology can overcome these challenges. For example, the combination of multispectral imaging and NDVI can significantly improve the estimation accuracy of LAI for urban parks [15]. UAVs can collect high-resolution multispectral images through low-altitude flight, allowing researchers to analyze the LAI of urban parks in detail, achieving significantly higher accuracy than traditional satellite remote sensing technology [16]. Additionally, the flight time and path flexibility of UAVs enable researchers to obtain the latest vegetation data at any time, thereby avoiding the data latency issues associated with satellite remote sensing technology, which can be caused by long time intervals or poor weather conditions [17].

Compared to traditional linear regression or simple nonlinear regression models, machine learning models such as Back Propagation Neural Network (BPNN) and Random Forest (RF) are more flexible and powerful. Among them, RF is widely applied in the field of LAI inversion. As a decision tree-based ensemble method, RF can effectively capture the complex nonlinear relationship between NDVI and LAI by constructing multiple decision trees, thereby improving the model’s fitting accuracy [18]. In addition, it generates branches for each decision tree by randomly selecting samples and features, which effectively reduces the overfitting risk and demonstrates strong generalization ability when dealing with different vegetation types and spatial scales [19]. Another advantage of RF is its robustness to uncertainty and noisy data in NDVI and LAI inversion. By integrating the prediction results of multiple decision trees, RF can estimate LAI more stably, especially in high-density or spatially heterogeneous vegetation areas, where its prediction accuracy is significantly higher than that of traditional regression methods [20]. The stability and high accuracy endow RF with broad application potential in LAI assessment of complex urban environments, providing more reliable technical support for urban green space management and ecosystem service optimization.

**Fig 1:**
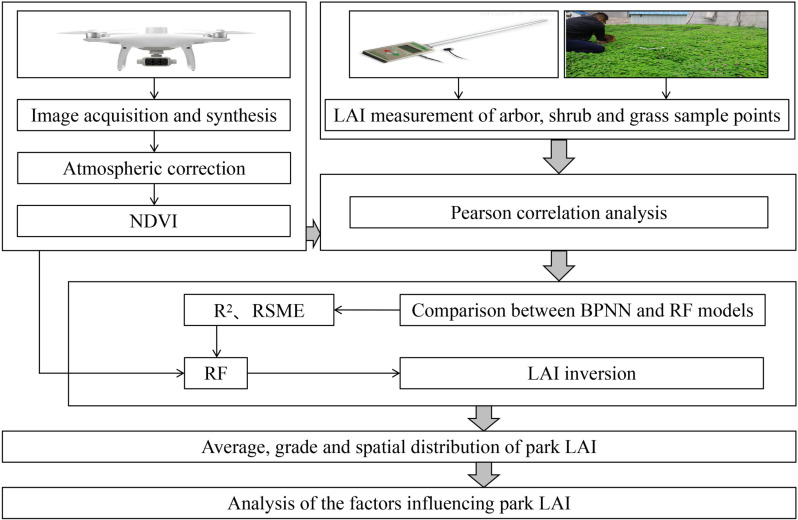
Research framework.

This study aims to perform LAI inversion of urban parks based on NDVI using RF. First, high-resolution remote sensing images are obtained using multispectral sensors mounted on a UAV and NDVI is calculated. Next, LAI data of the sampling points is collected through ground measurement. A training dataset is then established and used to construct the RF model, in order to identify the nonlinear relationship between NDVI and LAI. Subsequently, a refined image reflecting the distribution of LAI within the park is generated using the trained RF. Finally, data support and decision-making references are provided for the management and optimization of urban green spaces through an in-depth analysis of LAI characteristics. The research framework is shown in Fig 1.

## Materials and methods

### Study area

**Fig 2:**
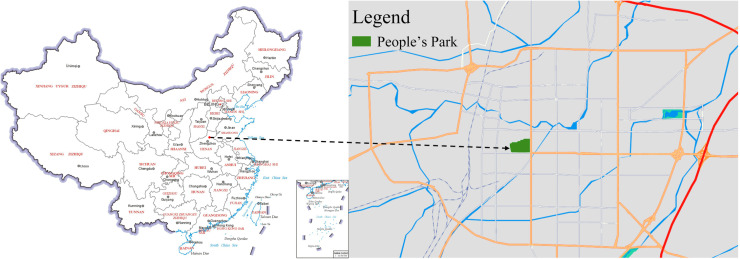
Location of Xinxiang people’s park (map sourced from the Ministry of Natural Resources of China, Carto-graphic License: GS (2019)1676).

Xinxiang People’s Park is located in Xinxiang City, northern Henan Province, China, in the plain region of the middle and lower reaches of the Yellow River. Its geographical coordinates range from 113°31’ E to 114°49’ E and from 34°53’ N to 35°51’ N (Fig 2). Xinxiang People’s Park is a large and comprehensive urban park with a long history. It covers an area of approximately 40 hectares and was established in the 1950s, making it nearly 80 years old. The park features high vegetation coverage, with a rich vegetation structure and diverse plant species. After years of development, many trees have reached maturity, forming a relatively stable vegetation system. Additionally, the park’s vegetation undergoes significant seasonal changes, offering a unique landscape in each season. With its rich plant communities, beautiful landscapes and a long history, People’s Park has become one of the most representative parks in Xinxiang City.

### Data source and preprocessing

#### Acquisition and processing of multispectral image data.

The UAV DJI Phantom 4 Multispectral (DJI Innovations Co., Ltd., Shenzhen, China), was used to capture images. It was equipped with a 2-megapixel multispectral camera with the following band and wavelength information: Blue (B): 450nm ±  16nm; Green (G): 560nm ±  16nm; Red (R): 650nm ±  16nm; Red Edge (RE): 730nm ±  16nm; Near-infrared (NIR): 840nm ±  26nm. The flight took place on August 9, 2022, under cloudless skies and optimal lighting conditions. The UAV flew autonomously according to preset flight parameters and route, at a height of 200m above the ground, with a forward overlap of 80%, a side overlap of 80%, and spatial resolution of 10.74 cm. A total of 447 images were captured for each waveband during the flight, and Pix4D mapper 4.4 software (Pix4D SA, Lausanne, Switzerland) was used for image stitching.

The DJI Phantom 4 Multispectral ensured spatial accuracy through its integrated Real-Time Kinematic (RTK) system, providing positioning with a horizontal accuracy of 1 cm + 1ppm and a vertical accuracy of 1.5 cm + 1ppm, enabling high-precision positioning without the need for ground control points [21,22]. A multispectral light intensity sensor was integrated at the top of the UAV, and the solar irradiance data can be used for light compensation of the images. This step removed the interference of ambient light during data acquisition, and significantly improved the accuracy and consistency of data collected over different time periods, eliminating the need for whiteboard calibration. The selection of flight time was based on the solar elevation angle recommended by DJI GS PRO software, minimizing data errors caused by changes in the solar elevation angle [23]. Atmospheric correction was performed using QUAC (Quick Atmospheric Correction) tool in ENVI 5.3 (L3Harris Geospatial Solutions, Westminster, CO, USA).

#### Calculation and processing of NDVI for park vegetation.

The calculation formula for NDVI is as follows [24]:


$$\text{NDVI =}\frac{\text{NIR}-\text{RED}}{\text{NIR + RED}}$$ (1)

Where NIR is the reflectivity of the near-infrared waveband, and RED is the reflectivity of the red band.

When NDVI is close to 0 or negative, it indicates non vegetation areas such as bare soil, rocks, and urban areas.

#### Measurement of vegetation LAI of park.

**Fig 3:**
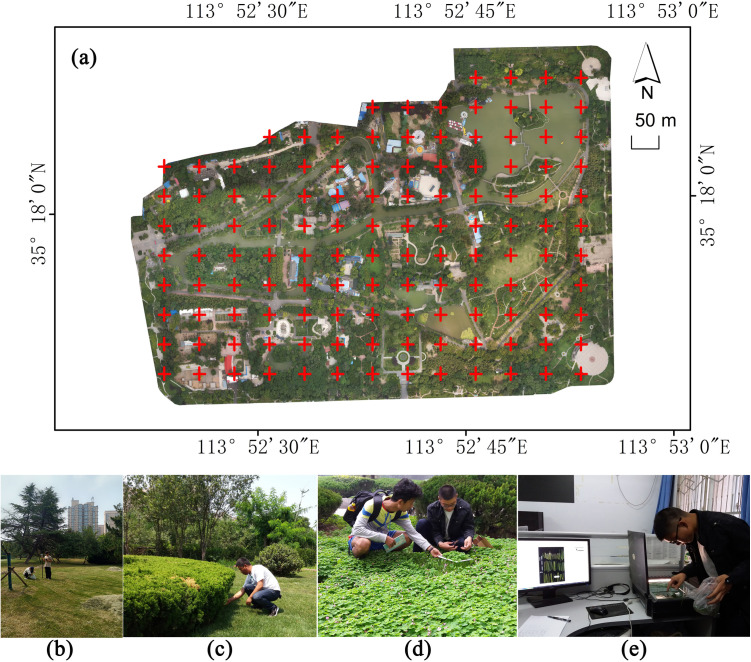
(a) Sample distribution; (b) RTK positioning of sampling points; (c) Measurement of shrub LAI; (d) Measurement of grass LAI; (e) Scan the grass leaf area.

ArcGIS 10.5 software (Environmental Systems Research Institute, Inc., Redlands, CA, USA) was used to mesh the images of People’s Park, and 125 initial sampling points were obtained. After removing samples located on roads and water surfaces, 114 valid samples from the vegetation coverage areas were retained. The coordinates of the valid samples were extracted and exported as data point files. These data point files were then imported using the Situoli S5II RTK surveying instrument (Guangzhou Situoli Surveying and Mapping Technology Co., Ltd., Guangzhou, China). Following the instrument’s instructions, researchers pinpointed the corresponding ground positions of each sampling point (Fig 3).

**Fig 4:**
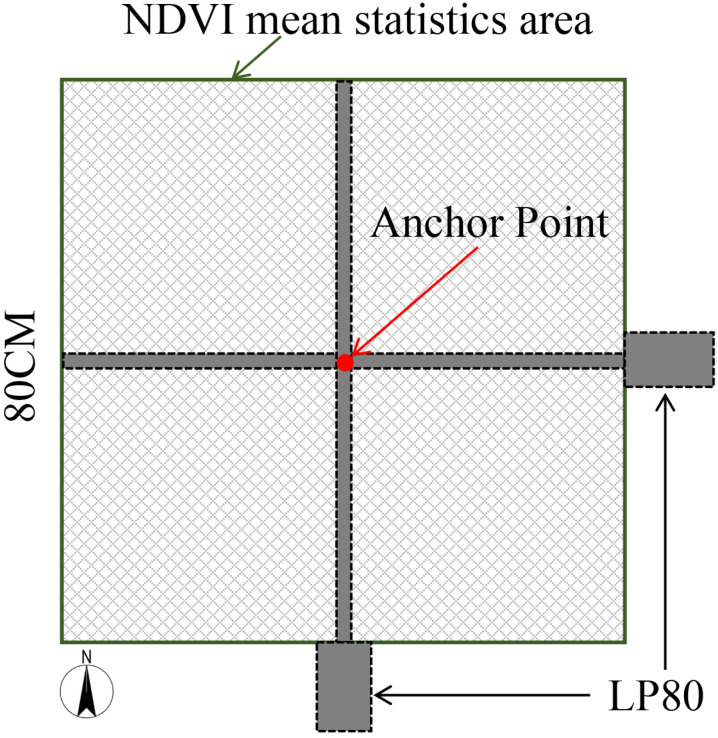
NDVI statistical area for arbor and shrub sampling points and LAI measurement method.

The ACCUPARmodelLP-80 (METER Group, Inc., Pullman, WA, USA) portable LAI measuring instrument was used to measure LAI at each arbor and shrub sampling point. With an LP-80 receiving rod length of 0.8 m, an area of 0.64 m² was designated as the sampling zone, with each sampling point at the center. LAI measurements were conducted between 10:30 a.m. and 2:00 p.m. on August 19 and 20, 2022. During this time, the solar zenith angle was at its smallest, improving the accuracy of the measurements. Within each sampling area, LP-80 measurements were taken five times in the north-south and east-west directions along the rectangle passing through the centerline, and the mean value was then calculated. To ensure consistency in data collection, ArcGIS 10.5 software was used to calculate the average NDVI value within a square area (Fig 4).

The LAI of grass vegetation was determined using the weighing method. A 20 cm ×  20 cm square frame was placed on the grass. Leaves outside the frame were first trimmed to avoid interference, and then the leaves inside the frame were collected and placed into a bag for transportation to the laboratory.

The entire bag of leaves was weighed using a centesimal balance (M_T_), and a subset leaves were selected for individual weighing (M_P_) before scanning. First, an Epson A3 format scanner (Epson China Co., Ltd., Beijing) was used to scan the leaves after they were neatly arranged. Next, Photoshop software was employed to perform binarization on the scanned images. Finally, leaf area statistics (S_P_) were obtained using ImageJ software (National Institutes of Health, MD, USA) to calculate LAI_grass._


$${\text{S}}_{\text{T}}\text{=}{\text{M}}_{\text{T}}{\text{/M}}_{\text{P}}\text{×}{\text{S}}_{\text{P}}$$ (2)

$${\text{LAI}}_{\text{grass}}\text{=}{\text{S}}_{\text{T}}\text{/0}{\text{.04 m}}^{\text{2}}$$ (3)

### Model construction and LAI inversion

#### Model construction.

NDVI and LAI data were imported into SPSS software (International Business Machines Corporation, NY, USA) for correlation analysis. The correlation coefficient was 0.332, indicating that there is no significant linear relationship between NDVI and LAI. This suggests that the relationship between NDVI and LAI may be complex and cannot be accurately fitted using traditional regression methods.

Python (Python Software Foundation, OR, USA) was then used to construct RF to further analyze the relationship between NDVI and LAI. NDVI was selected as the independent variable, while LAI was chosen as the dependent variable. The 114 sample data points were divided into a training set and a testing set, with 70% of the data used for training and 30% for testing. The model’s performance was evaluated using the test set data, and the R-squared (R²), Mean square Error (MSE), and Root Mean Square Error (RMSE) of the model were calculated. R² represents the proportion of the total variation explained by the model, while MSE is used to calculate the average sum of squared differences between predicted and actual values. The formula is:


$$\text{MSE}={\text{σ}}^{\text{2}}\text{×}\left(\text{1}-{\text{R}}^{\text{2}}\right)$$ (4)

RMSE is the square root of MSE, and is used to convert the error measure back to the same unit as the original data for interpretation and comparison. The formula is:


$$\text{RMSE =}\sqrt{\text{MSE}}$$ (5)

The parameters for the BPNN model are as follows: hidden_layer_sizes =  (200, 100, 50), max_iter =  5000, activation =  relu, learning_rate =  adaptive, random_state =  42, alpha =  0.0001. The results yielded an R² of 0.18 and an RMSE of 1.35. The parameters for the RF model are: n_estimators =  1000, max_depth =  20, min_samples_split =  5, min_samples_leaf =  5, max_features =  sqrt. The results yielded an R² of 0.54 and an RMSE of 0.91. A comparison indicates that the RF model demonstrates higher accuracy.

#### LAI inversion.

The calibrated and mosaicked multispectral imagery was imported into ArcGIS 10.5 software. The NDVI raster image for the park was generated using the band calculation tool. The pre-trained RF model was loaded in Matlab 2021 (The MathWorks, Inc., MA, USA) to read the NDVI image and obtain the LAI, with the MAT format converted to CSV format. Next, Python was used to convert the CSV format into TIF format. Finally, the TIF image was imported in ArcGIS 10.5, followed by reclassification, completing the statistical analysis and mapping of LAI.

## Results

**Fig 5:**
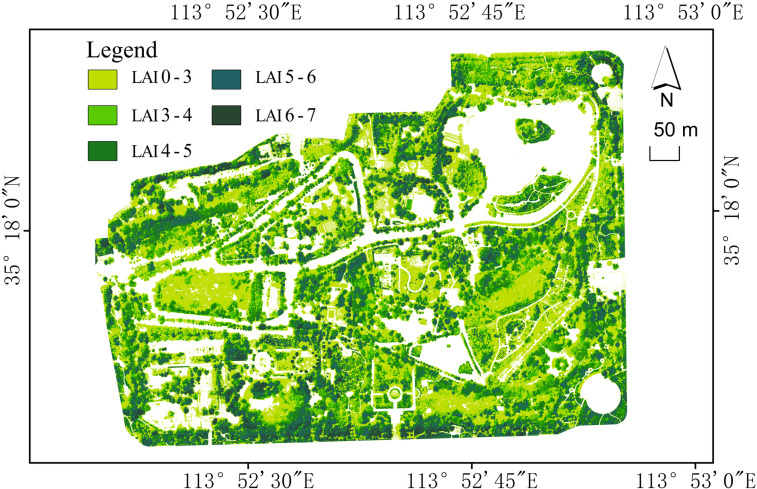
LAI inversion of Xinxiang People’s Park.

The inversion results are shown in Fig 5.

### Average of park’s LAI

The average LAI of a park is an important indicator for measuring vegetation coverage and ecological health. It reflects the contribution of vegetation functions to ecosystem services such as carbon sequestration, water regulation, and air purification, etc. Research has shown that the average LAI of Wuhan City Park ranges from 1.8 to 2.0, indicating good vegetation coverage and health status within the park. LAI provides a basis for the ecological function assessment of urban green spaces and is especially crucial for enhancing urban climate regulation and ecosystem services [25]. Research on Hefei Huancheng Park indicates that the average LAI of the city’s forest community is 1.95, reflecting its good ecological health status as an urban forest. A high LAI suggests that these urban forests play an important role in providing ecosystem services such as carbon sequestration and air quality improvement [26].

The average LAI of Xinxiang People’s Park is 2.30 (the average of the entire park), and after excluding the hard areas such as buildings, squares, and roads (which accounting for 36.05% of the park), the average LAI increases to 3.59. After 80 years of development, the vegetation in the park has fully matured, with a significant increase in tree crown expansion and vegetation density, which are key factors contributing to the park’s high LAI. Mature trees and shrubs form a multi-layer vegetation structure, effectively filling the vertical space and significantly increasing the total leaf area. Additionally, the park features a diverse plant community, including trees, shrubs and grass. This multi-layered and multi-species vegetation configuration not only enhances biodiversity but also increases the park’s LAI. Long-term, refined management practices are also a crucial factor affecting the park’s LAI. Regular maintenance measures such as soil improvement, irrigation, and pruning help maintain the healthy growth and expansion of plants, ensuring that the park retains its high ecological functions over time.

### Grade distribution of park’s LAI

The grade distribution of the park’s LAI is shown in Table 2. Generally, the low LAI (<3) interval accounts for a high proportion of 42.85%, indicating that a considerable number of areas in the park have low vegetation coverage, resulting in relatively low ecological benefits and carbon sequestration capacity. These areas may be grasslands, shrubs, or sparse vegetation. The medium LAI interval (3-5) is more concentrated, with 3-4 range accounting for 17.59% and 4-5 range accounting for 20.42%, totaling approximately 38.01%. This interval typically represents vegetation with moderate coverage density, such as moderately dense trees or high shrubs, which provide moderate ecological services for the park, such as shading and humidity regulation. There are fewer areas with high LAI, with the 5-6 and 6-7 intervals accounting for 15.21% and 3.93%, respectively, totaling 19.14%. These areas are likely to be forests or dense tree regions with high vegetation density. Although the proportion of such areas is relatively small, they usually provide higher ecological functions, including greater carbon storage and ecological stability.

**Table 2:** LAI grade distribution of Xinxiang People’s Park.

No.	Grade	LAI interval	Proportion(%)
1	Low	<3	42.85
2	Medium	3–4	17.59
3		4–5	20.42
4	High	5–6	15.21
5		6–7	3.93

### Spatial distribution of park’s LAI

From a spatial distribution perspective, high LAI areas are concentrated around the periphery of the park. Dense trees and tall plants effectively block the line of sight between the interior and exterior areas of the park, reducing visual interference from the outside and providing a more enclosed and private space for visitors. This design makes people feel closer to nature while isolating the hustle and bustle, as well as the visual pollution of the city [27]. The tall trees and plants on the periphery can serve as the “background” in landscape design, creating a contrast with the vegetation inside the park and enhancing the layer and depth. When tourists enter the park, they gradually transit to the open landscape area through the barrier plants on the park’s periphery, increasing the variability and richness of the space. Medium LAI areas are typically found in semi-open spaces with relatively less human activity within the park. The vegetation here is usually denser than in lawns and sparse forests, but the distribution of trees is not as compact as in dense forests. These areas are typically consist of shrubs, grasslands, and a small number of trees, where the tree crowns are partially covered but not fully dense, allowing sunlight to enter. The tree crown layers are relatively few, usually consisting of a single layer or a small number of layers, unlike the complexity of dense forests [28]. Low LAI areas are concentrated in regions with high activity frequency, such as lawns and sparse forests. The planting density in these areas is usually artificially controlled, and plant coverage is limited, resulting in low LAI values in these regions [29].

## Discussion

### Factors influencing park’s LAI

#### Impact of vegetation types on park’s LAI.

In 2017, the research team conducted an LAI survey at People’s Park in Xinxiang City and found that the LAI of most plants reached its peak between July and August. The LAI intervals were as follows: deciduous arbor (2.1-6.16), evergreen arbor (2.27-4.86), deciduous shrub (3.94-5.86), evergreen shrub (4.61-6.57), deciduous hedge (4.02-6.53), evergreen hedge (4.89-6.91), and lawn (1.92-7.83), showing significant differences [30].These differences are mainly due to variations in leaf shape, canopy structure, and leaf area density among different plant species, which directly affect the biomass and LAI of the tree crowns. People’s Park has a rich diversity of plant species, with 156 species belonging to 59 families and 114 genera [31]. The increase in species diversity positively impacts LAI, thereby enhancing the productivity and stability of ecosystems [32]. The diverse vegetation configuration not only enhances the ecological value of the park, but also provides more stable ecological service functions for the city.

#### Influence of vegetation configuration pattern on park’s LAI.

The combination and vertical distribution structure of different species in the park significantly impact the size and distribution of LAI. The vegetation configuration of the People’s Park is diverse, mainly consisting of tree-shrub-grass, tree-grass and shrub-grass, etc. Some areas have developed relatively mature community structures. The park can be divided into different zones based on functional uses and landscape viewing needs through various combinations of trees, shrubs, and grasses. In this way, the park not only provides rich ecological benefits, but also meets the recreational, leisure, and fitness needs of visitors. The richness of vegetation layers not only enhances the park’s ecological function but also increases its visual depth and layering. Tall trees contrast sharply with low shrubs and grass, creating a varied landscape effect.

There are differences in the contribution of different canopy levels (such as tree layer, shrub layer, and grass layer) to LAI. Multi-layered vegetation systems with complex structures usually have higher LAI, which helps maintain the stability and productivity of ecosystems [33]. The combination of trees and shrubs significantly improves LAI under the mixed planting mode, yielding better results than communities with a single plant species. This multi-layered canopy structure not only increases LAI but also effectively alleviates the urban heat island effect, with a significant impact on reducing urban temperatures, especially in summer [26].

#### Impact of planting density on park’s LAI.

In park design, different functional areas are typically divided by adjusting planting density to meet the diverse activity needs of the public, with common types including sparse forest and grassland areas, as well as dense forest areas. The vegetation density in sparse forest and grassland areas is relatively low, typically resulting in lower LAI values, making these areas more suitable for open activities. However, other functional areas, aside from sparse forest and grassland, can increase LAI values by increasing planting density. High density vegetation, especially trees with large crown widths, has a significant cooling effect on the environment. These trees can substantially reduce air temperature by increasing shading area and transpiration, thereby enhancing the cooling effect of the local environment [34]. In urban environments, densely planted trees can not only increase the LAI of green spaces, but also enhance their ecological service functions, such as air purification, cooling, and carbon sequestration, etc [35]. Therefore, reasonable adjustment of planting density of trees in park planning and design can not only optimize the LAI value, but also effectively improve the ecological benefits of the park.

### The applicability of UAV and RF for LAI inversion in parks

#### The applicability of UAV for LAI inversion in parks.

In traditional urban green space research, remote sensing images typically have the following resolutions: 30 m for TM images, 2.5-10m for SPOT5 images, and 1m for IKONOS-2 images [36,37]. Those low-resolution images prevent remote sensing data from accurately reflecting the characteristics of individual surface features, thereby affecting the accuracy of LAI estimation. In contrast, the resolution of multispectral images used by the UAV in this study is 10 cm, allowing for more accurate monitoring of vegetation status in local green spaces and helping to identify smaller-scale ecological changes. The high spatial resolution of UAV multispectral imaging enables it to capture subtle vegetation changes in urban green spaces with greater precision. Compared to traditional satellite remote sensing data, the high-resolution data obtained by UAVs can avoid the issue of mixed pixels. For LAI estimation in urban green spaces with high-density vegetation or complex structures, UAV images with a resolution of 1 m or less are necessary [16].

#### The applicability of RF for LAI inversion in parks.

In this study, for the RF model: R² =  0.54, RMSE =  0.91, which indicates that the fitting accuracy is lower compared to other existing studies. This is because the vegetation type in farmland and grassland are more homogeneous, and the degree of vegetation overlap is lower, leading to better inversion performance. Wang et al. evaluated the effectiveness of machine learning method in estimating the LAI of rice field, and RF showed the best accuracy with a test set of R² = 0.84 and RMSE = 0.67 [38]. Darvishzadeh et al. used the PROSAIL model to invert the LAI of grassland based on NDVI, achieving R² = 0.89 and RMSE = 0.22 [39]. After further improving the model, R² reached 0.91, indicating that combining physical and statistical models can significantly enhance the accuracy of LAI inversion. Li et al. obtained RMSE = 0.1956 when using RF to predict the LAI of grassland, demonstrating the strong capability of RF in grassland LAI prediction [40]. Forests, however, have a diverse vegetation distribution and a significant vertical structure, which complicates the inversion process. Srinet et al. used RF to estimate LAI in tropical forests and obtained R² = 0.79 and RMSE = 0.14, demonstrating excellent model performance in estimating LAI. Additionally, its prediction accuracy can be further improved by selecting the best predictive variable [41]. In the study by Gonsamo et al., the results of using NDVI to invert LAI in forests showed R² = 0.63 and RMSE = 0.52 [42].

Urban parks place high demands on both ecological functionality and spatial design. To meet these requirements, designers often use different vegetation configurations, such as tree-shrub-grass, tree-grass, shrub-grass, and lawns. Additionally, to enhance the aesthetic appeal, designers design the vegetation patterns with different colors, textures, and leaf shapes. In some cases, non-native species may also be chosen. Vegetation is often pruned regularly to maintain the desired landscape effect, which alters its natural growth pattern or growth cycle. These combined factors influence the growth and ecological balance of vegetation, making its response to environmental changes more complex and unpredictable.

### Limitations and future work

#### Limitations.

Although this study has yielded promising results, it still faces some challenges and limitations. Firstly, while UAV-captured images have high resolution, the data acquisition process can be influenced by factors such as weather conditions, flight altitude, and camera calibration, which may degrade image quality and ultimately affect the accuracy of LAI inversion. In complex urban environments, buildings, shadows and the mixture of different vegetation types may interfere with remote sensing data, thereby affecting the model’s fitting performance. Secondly, errors in ground measurement data represent another significant source of uncertainty. In this study, ground-based LAI measurement data serve as an important reference for model training, but the data are often limited by the number and distribution range of sampling points, which may not fully capture the vegetation characteristics of the entire study area. Systematic errors or insufficient sampling in the ground measurement data may lead to deviations in the model results.

Unlike farmland and grasslands, which typically have single-species and relatively uniform distribution, and unlike naturally grown forests with complex structures, park vegetation is entirely shaped by the designer’s decisions. As a result, the LAI distribution in parks is a complex outcome influenced by the designer’s subjective choices, natural growth, and human management. Currently, our analysis focuses on tree species and structure, but a more in-depth study will require additional data on forest structure.

The fitting accuracy of NDVI and LAI may vary for different vegetation types with distinct growth forms, such as trees, shrubs, and grass. If classification-based fitting and inversion were applied, the results could potentially be more precise. However, parks feature a rich diversity of plant species, varied spatial arrangements, and complex vertical structures, with trees, shrubs, and grass interwoven into a complex system. Currently, it is challenging to clearly differentiate their proportions and distributions, which somewhat impacts inversion accuracy. Additionally, NDVI mainly reflects the coverage and health status of ground-level vegetation, and it cannot comprehensively capture the growth characteristic differences among various vegetation types. Meanwhile, other vegetation indices, such as the Enhanced Vegetation Index (EVI), Soil Adjusted Vegetation Index (SAVI), and Green Normalized Difference Vegetation Index (GNDVI), may offer higher fitting accuracy.

#### Future work.

To further improve the accuracy and applicability of LAI inversion, future research should combine other remote sensing technologies or models for comparative analysis. On one hand, future studies should aim to combine UAV multispectral images with other remote sensing data (such as hyperspectral images and LiDAR data), and leverage the complementarity of multi-source data to enhance LAI inversion accuracy. For example, LiDAR data can provide information on vegetation height and three-dimensional structure, offering a more comprehensive reflection of vegetation’s physical characteristics when combined with multispectral images. On the other hand, regarding modeling methods, researchers could compare RF with other machine learning models, such as support vector machines and neural networks, to evaluate the performance differences in LAI inversion.

By utilizing the distinct textural characteristics of trees and grass, it may be possible to differentiate between these two vegetation types. Performing separate LAI inversion for trees and grass could potentially improve result accuracy. Additionally, future research could focus on incorporating time-series remote sensing data to further optimize the LAI inversion model by analyzing the multi-temporal vegetation change trends. This approach not only enhances the dynamic response capability of the model, but also demonstrates higher accuracy and stability when accounting for seasonal changes. In general, future work should continue to combine multi-source remote sensing data and advanced modeling techniques to further improve the accuracy of urban park ecological function assessment and provide a more scientific basis for urban green space management.

## Conclusions

(1) The average LAI of Xinxiang People’s Park is 2.30 for the entire park. After excluding the hard areas, such as buildings, squares, and roads (which account for 36.05%), the average LAI increases to 3.59, indicating good vegetation conditions. In terms of spatial distribution, high LAI areas are concentrated around the periphery of the park, where dense trees and lakes are located, while low LAI areas are primarily found in lawns and areas with high human activity. The analysis of LAI distribution provide scientific basis for green space management and vegetation configuration in urban parks.
(2) The LAI of parks and its distribution are influenced by factors such as vegetation type, spatial arrangement, planting density, landscape aesthetics, and site functionality.
(3) Based on high-precision UAV multispectral data, this study used NDVI and ground LAI measurement from 114 sampling points in Xinxiang People’s Park for RF analysis. Although the model’s fitting accuracy is still less than ideal, the advantages of RF in handling nonlinear relationships make it an effective tool for LAI inversion in complex vegetation environments.
(4) As an indicator of vegetation surface coverage, NDVI has certain limitations in representing the growth characteristics of different vegetation types. Future research could integrate multi-source remote sensing data, such as hyperspectral imagery or LiDAR data, to improve the accuracy of LAI inversion.
